# Personalizing ecological momentary intervention for substance use disorders through data-driven decision rules

**DOI:** 10.3389/fpsyt.2026.1717544

**Published:** 2026-03-02

**Authors:** Mina Kwon, Joo Yun Song, Jae Yeon Hwang, Su Jeong Seong, Kee Jeong Park, Young Tak Jo, Yeo Jin Kim, Moo Eob Ahn, Sang-Kyu Lee

**Affiliations:** 1 Department of Psychiatry, Kangdong Sacred Heart Hospital, Hallym University College of Medicine, Seoul, Republic of Korea; 2 Department of Neurology, Kangdong Sacred Heart Hospital, Hallym University College of Medicine, Seoul, Republic of Korea; 3 Department of Emergency Medicine, Chuncheon Sacred Heart Hospital, Hallym University College of Medicine, Chuncheon, Republic of Korea; 4 Department of Psychiatry, Chuncheon Sacred Heart Hospital, Hallym University College of Medicine, Chuncheon, Republic of Korea

**Keywords:** ecological momentary intervention (EMI), personalized intervention, real-time intervention, real-time risk detection, substance use disorders (SUDs)

## Abstract

Substance use disorders (SUDs) are highly prevalent and lethal, yet treatment reach remains below 20%. As risk of substance use and relapse is episodic and context-dependent, ecological momentary interventions (EMIs) that deliver real-time intervention in daily life are promising, but findings to date remain mixed. We argue this variability reflects the importance of decision rules, when to deliver which intervention. However, current EMI systems mostly rely on static, one-size-fits-all rules that could not account for between-person differences and within-person fluctuations. We suggest a data-driven approach for building EMI systems, aiming to better address the heterogeneity of SUDs. First, collect multimodal, multicontextual data—spanning controlled laboratory tasks, everyday smartphone and wearable signals, and periods when devices are offline—to complement blind spots of individual data sources. Next, build context−aware prediction models that estimate momentary risk and validate predictors across contexts and modalities, enabling features discovered in one setting to be translated into signals available in another. Finally, implement real−time, context−sensitive decision rules that best fit the contextual profile of the risk. By centering EMIs on explicit, testable decision rules, this approach will offer a practical path to reducing variability in outcomes and deliver more reliable, personalized support at the moments and places where risk emerges.

## Introduction

Substance use disorders (SUDs) are among the most prevalent and deadly psychiatric disorders. The lifetime prevalence of SUDs is estimated at approximately 35% (1, 2). Harmful alcohol use is linked to about 2.6 million deaths annually worldwide, with psychoactive drug use contributing an additional 0.6 million deaths (3). Furthermore, cigarette smoking accounts for roughly 8 million deaths each year (4). Despite this burden—and the demonstrated effectiveness of traditional treatments such as pharmacotherapies, Alcoholics Anonymous, cognitive behavioral therapy (5, 6) or Motivational Interviewing (7)—the global proportion of individuals with SUDs who receive treatment remains below 20% (8–11). This treatment gap has been commonly accounted for public stigma, lack of insight, but is also due to time and regional barriers (12–15).

Closing this gap requires interventions that reach people in their daily lives and respond in real time to emerging risk, because the risk of substance use is episodic and context-dependent. Specifically, the risk of substance use and relapse reflects proximal states such as craving, stress, withdrawal, and cue exposure. Of these, craving, a “strong desire for drugs” (16), has been suggested as an important underlying mechanism and proximal predictor for drug use and relapse (17–19). However, craving is a complex and subjective phenomenon, and is strongly influenced by internal factors and environmental contexts (20, 21). Effective care therefore requires timely support that aligns with the moments and contexts in which risk emerges. This motivates the use of ecological momentary intervention (EMI)—treatments delivered in people’s everyday lives in a timely manner.

EMI was introduced in 2005 as interventions tailored to ecological momentary assessments (EMA)—brief, repeated self−reports collected in daily life—to tailor support in real time (22). However, terminology and definitions vary across the literature (23). In this review, we follow (24) and define EMI as treatments delivered in real time in real-life settings. This definition does not necessarily require EMA to be embedded in EMI, and embedding an intervention in technology such as mobile application alone does not meet EMI criteria unless it aims to deliver timely, *in situ* support. Just-in-time adaptive intervention (JITAI) is treated as a subtype of EMI, the core difference lying in whether EMI adapts to each individual’s momentary state and context (25).

Evidence for EMIs in SUDs is promising yet mixed, reflecting heterogeneity in their designs, study populations, participant engagement, and outcome variables (26–28). However, these mixed findings do not imply that EMIs lack efficacy; rather, they suggest that effectiveness is conditional on decision rules, including when an intervention is delivered, which specific intervention is chosen, and through which modality it is delivered. We therefore shift the question from “Do EMIs work” to “Under which decision policies do EMIs work?”

In response, we outline a data−driven framework that can be tuned across conditions and contexts to align timing and content with individual and situational risk. Accordingly, we (i) briefly review the current status of EMIs for SUDs, (ii) critically evaluate existing decision rules for timing and intervention selection, and (iii) outline a pragmatic, data−driven, context−aware framework for developing personalized EMI for SUDs, focusing on decision policies to guide both when and what to deliver.

## Overview of ecological momentary interventions for SUDs

EMIs for SUDs typically target abstinence or relapse prevention and often model proximal risk such as craving or urge. Common outcome variables include biochemically verified abstinence, time−to−lapse, days used, and consumption intensity. In practice, EMIs span (a) EMA−linked systems that use short self−reports to detect risk and (b) sensor−linked systems that leverage passive smartphone/wearable data to infer context and state; both deliver *in situ* interventions in real time. With the shift to smartphones and wearables, mobile apps have become the dominant delivery channel.

Across substances, results from previous randomized controlled trials (RCTs) are promising but mixed (**Table 1**). In tobacco, a meta-analysis of nine RCTs of smartphone-based cessation EMIs found no overall effects on abstinence, although a significant benefit was observed when EMIs were combined with pharmacotherapy (26). In individual RCTs using more context-aware delivery rules, Global Positioning System (GPS) geofencing–triggered support was associated with higher 6-month cotinine-verified sustained abstinence compared with usual care (29), and an EMA-informed, risk-profile–tailored EMI delivered alongside nicotine replacement therapy increased abstinence at 26 weeks in a low-income sample (30). Alcohol-focused trials similarly span modest benefits to null findings. For example, an early small RCT among college students reported fewer drinks per day but no differences on other drinking outcomes (33), whereas a larger trial among adults leaving residential treatment reported fewer risky drinking days at months 4 and 12 (but not month 8) using GPS-triggered alerts (34). In contrast, an RCT delivering tailored messages during drinking occasions showed small effects with no significant between-group differences on peak risky single-occasion drinking (35), and a large trial adding event-based EMI texts to a web-based intervention around high-risk university events found no group differences in weekend drinking (37). Other alcohol EMIs that delivered scheduled, tailored messages to support reduced drinking also reported mixed findings across cohorts (36). Beyond alcohol and tobacco, adding Addiction-Comprehensive Health Enhancement Support System (A-CHESS) to medication treatment for opioid use disorder did not significantly improve validated abstinence from illicit opioids (38), while a multi-substance trial in treatment settings reported a small but statistically significant increase in days abstinent among participants receiving EMI components (39).

**Table 1:** Overview of ecological momentary interventions for SUDs.

Authors	Target substance	Study design	Sample (analyzed)	Ecological momentary intervention (EMI)	Primary outcome	Effectiveness of EMI
(29)	Tobacco	Two-arm RCT	Smokers willing to set a quit date within one month (n = 209) - usual care (n = 105) - usual care + EMI (n = 104)	GPS geofencing-triggered text messages	6-month biochemically verified sustained abstinence (cotinine-verified)	Biochemically verified sustained abstinence was higher in the EMI group than in the control group
(26)	Tobacco	Meta-analysis	9 RCTs of smartphone interventions for smoking cessation (n = 12,967)	Smartphone app-based interventions to support smoking cessation	Smoking abstinence rate	No overall effect of apps versus control on abstinence, but a significant benefit when combined with pharmacotherapy
(30)	Tobacco	Two-arm RCT	US adults with household incomes below 200% of the federal poverty line (n = 454) – NCI free smoking cessation app + nicotine replacement therapy (n = 229) – automated smartphone-based EMI + nicotine replacement therapy (n = 225)	Messages selected from a library of >400 expert-reviewed messages based on each participant’s EMA-derived risk status and trigger profile (31, 32)	Biochemically verified 7-day point prevalence abstinence at 26 weeks post-quit (CO verification)	Abstinence rate was higher in the EMI group than in the control group
(33)	Alcohol	Two-arm RCT	College students who reported drinking more than once per week (n = 40) - daily survey only (n = 20) - daily survey + EMI (n = 20)	EMA-triggered text messages stratified into three conditions (negative consequences from drinking; drinking without consequences; not drinking)	Alcohol use and consequences over 2 weeks (total drinks; drinking days; drinks/day; negative consequences)	The EMI group reported fewer drinks per day than the control group, with no significant differences on other outcomes
(34)	Alcohol	Two-arm RCT	Adults meeting DSM-IV criteria for alcohol dependence entering residential treatment (n = 349) – treatment as usual (n = 179) – treatment as usual + EMI (n = 170)	GPS geofencing–triggered text messages when entering prespecified high-risk locations	Risky drinking days in the past 30 days	The EMI group reported fewer risky drinking days than the control group at months 4 and 12, but not at month 8
(35)	Alcohol	Three-arm RCT	Young adults screened for risky single-occasion drinking (≥5 drinks on one occasion in the past 3 months; n = 269) - control (n = 90) - EMA-only (n = 89) - EMA + EMI (n = 90)	Tailored text messages reinforcing motivations/intentions, offering harm-reduction tips, and providing feedback on cumulative drinking based on EMA responses	Number of standard drinks consumed at the most recent peak risky single-occasion drinking event	EMI effects on drinking were small; no significant between-group differences
(36)	Alcohol	Two-arm RCT	Study 1: Mandated undergraduates who violated campus alcohol policy (screened positive for past-month alcohol use; n = 141) - in-person Brief Motivational Interviewing (BMI) (n = 70) - BMI + EMI (n = 71) [Study 2] Voluntary undergraduates (screened positive for past-month alcohol use; n = 238) - assessment only (n = 157) - BMI + EMI (n = 81)	Daily messages encouraging reduced drinking, tailored to participants’ motivation to change	Change in estimated peak Blood Alcohol Concentration (BAC) from baseline to the end of the 6-week intervention	Mixed findings across studies: Study 1 showed improved peak BAC in the EMI arm versus control; Study 2 showed no significant between-group difference in peak BAC (primary outcome), although secondary self-report outcomes improved
(37)	Alcohol	Three-arm RCT	Incoming first-year students (n =731) - assessment only (n = 241) - web-based intervention only (n = 246) - web-based intervention + EMI (n = 244)	Event-based text messages delivered around high-risk drinking events (e.g., orientation week and large university-run events), providing protective behavioral strategies and highlighting potential social consequences	Number of drinks per weekend during the first semester	No significant differences between groups
(38)	Opioid	Two-arm RCT	OUD patients meeting DSM-5 criteria for at least moderate severity, recruited from outpatient detoxification and treatment programs (n = 414) - medication for OUD (n = 206) - medication for OUD + EMI (n = 208)	A-CHESS (Addiction-Comprehensive Health Enhancement Support System), providing location-triggered alerts, self-monitoring, peer/counselor support, and tailored recovery messages	Self-reported abstinence from illicit opioid use during the past 30 days (validated by urine drug screens)	No significant differences between groups
(39)	Multiple (alcohol, stimulants, opioids, cannabis, other drugs)	Four-arm RCT	Adults from SUD treatment program (n = 401) - control (n = 98) - EMA only (n = 98) - EMI only (n = 100) - EMI + EMA (n= 105)	A-CHESS (Addiction-Comprehensive Health Enhancement Support System), providing location-triggered alerts, self-monitoring, peer/counselor support, and tailored recovery messages	Self-reported number of days abstinent from alcohol and other drugs in the past 90 days	A small but statistically significant effect of EMI on increasing the number of days abstinent

Studies were selected to represent ecological momentary intervention (EMI) research for substance use disorders (SUDs). To keep the table focused, we prioritized randomized controlled trials and one recent meta-analysis that evaluated real-time, *in situ* intervention delivery in daily life and reported substance use–related outcomes. Pilot or feasibility randomized trials were excluded. This selection was intended to be illustrative rather than exhaustive. RCT, Randomized Controlled Trial; EMA, Ecological momentary assessment; GPS, Global Positioning System; NCI, National Cancer Institute; CO, Carbon monoxide; OUD, Opiod Use Disorder.

Overall, findings to date are promising but mixed, making it difficult to draw firm conclusions about whether EMIs are effective across SUD populations and settings. In response to these limitations, recent work has increasingly applied machine learning to EMA and sensor streams to improve momentary risk prediction and enable more personalized, adaptive EMI strategies (40). This shift highlights that the key question is not only whether an EMI produced an effect, but how each system translated momentary data into intervention delivery in real time (41). Accordingly, to characterize current directions in the field, the next section reviews how prior EMI studies have operationalized intervention delivery, with particular attention to the decision rules that govern implementation.

## Limitations of the current decision rules in EMI for SUDs

Decision rules in EMIs can be grouped into two broad approaches: theory−driven and data−driven. In theory−driven approaches, established psychological or behavioral theories prescribe which data to monitor to detect risk, and what intervention to deliver. In data−driven approaches, these decisions are made based on empirical data and findings. However, even data−driven approaches are theory−informed when deciding which data to collect, and theory-driven approaches might include data-driven approach at some points, for example when validating theoretical predictions against empirical data. Acknowledging this grey area, we review prior EMI research based on its predominant approach (**Table 2**).

**Table 2:** Data-driven and theory-driven ecological momentary interventions for SUDs.

Authors	Target substance	Study design	Related theory	Data collection	When to deliver intervention	Which intervention to deliver	Effectiveness of EMI
(35)	Alcohol	Three-arm RCT	Motivational Interviewing	EMA self-report	- Event-based + time-based during drinking occasions: hourly messages from 7 pm to 2 am on drinking nights; feedback triggered by EMA entries	**[theory-driven]** Tailored text messages reminding participants of motivations/intentions, offering harm-reduction tips, and providing feedback on cumulative drinking based on EMA responses	Small effects; no significant between-group differences
(36)	Alcohol	Two-arm RCT	Motivational Interviewing	EMA self-report	- Fixed times	**[theory-driven]** Daily messages encouraging reduced drinking, tailored to participants’ motivation to change	Mixed: improved peak BAC in Study 1; no primary effect in Study 2 (secondary self-report improved)
(29)	Tobacco	Two-arm RCT	Incentive Sensitization Theory	EMA self-report; Passive data (geolocation, app interaction data)	- **[theory-driven]** Event-based: the app learns individual smoking locations/cues prior to the quit attempt and triggers support when users enter identified high-risk zones	Tailored support messages based on psychological and situational information users provide when marking personalized high-risk zones	Higher 6-month biochemically verified sustained abstinence vs control
(34)	Alcohol	Two-arm RCT	Self-Determination Theory; Incentive Sensitization Theory	EMA self-report; Passive data (GPS)	- **[theory-driven]** Event-based: GPS-triggered alerts when approaching high-risk locations - Fixed times	**[theory-driven]** A-CHESS (Addiction–Comprehensive Health Enhancement Support System) delivers continuing-care support and tailored motivational feedback using self-reported risk/protective factors (e.g., mood, sleep, time with family, Alcoholics Anonymous attendance) and location-based triggers	Fewer risky drinking days at months 4 and 12 (not month 8)
(42)	Any substance or behavioral addiction (trans-addiction; e.g., tobacco, alcohol, cannabis, stimulants, gambling, gaming)	Protocol for two-arm RCT	Incentive Sensitization Theory	EMA self-report; Passive data (GPS)	- **[theory-driven]** Event-based: triggered by user-reported craving/use/cues and GPS-detected at-risk locations - user-initiated	Context-matched interventions are delivered for each situation (use, craving, cues, at-risk location, or on-demand request), guided by the behavior change technique taxonomy (43)	N/A (study protocol)
(44)	Tobacco	Three-arm pilot RCT	N/A	EMA self-report	Event-based: delivered immediately after each EMA	**[data-driven]** After each EMA, the app computes a weighted lapse-risk score from the six predictors (31; 32): Risk score = 0.2(urge − 3) + 0.2(stress − 3) + 0.7(availability − 3) + social exposure(0/1) + alcohol use(0/1) − 0.2(motivation − 3). Messages are tailored to the predicted risk level (and dominant trigger)	No significant between-group differences in 12-week abstinence
(30)	Tobacco	Two-arm RCT	N/A	EMA self-report	Event-based (delivered immediately after each EMA); scheduled; user-initiated	**[data-driven]** Messages are selected from a library of >400 expert-reviewed messages based on each participant’s EMA-derived risk status and trigger profile (31, 32)	Higher biochemically verified abstinence vs control at 26 weeks

Studies are summarized according to their predominant decision-rule approach (theory-driven vs. data-driven). The table highlights how prior studies operationalized intervention delivery, including when interventions were delivered and how intervention content was selected. Brief summaries of EMI effectiveness are provided for orientation; detailed outcome definitions and results for efficacy-oriented randomized trials are presented in **Table 1**. This selection was intended to be illustrative rather than exhaustive. RCT, Randomized Controlled Trial; EMA, Ecological momentary assessment; GPS, Global Positioning System.

Most commonly, EMIs were developed based on theory-driven approaches, in which intervention timing and content are determined based on established psychological or behavioral theories. Several validated theories have been used to explain mechanisms of addiction and guide intervention strategies. For example, Motivational Interviewing is a widely used method for enhancing intrinsic motivation to change, such as quit drugs/alcohol, by exploring and resolving ambivalence (7). Drawing on this theory, prior EMI studies have delivered message content designed to enhance motivation not to drink alcohol—for example, by increasing self-awareness and reminding users of their personal goals (35, 36, 45, 46). Motivational Interviewing is conceptually aligned with Self-Determination Theory (47), which emphasizes competence, relatedness, and autonomy as core drivers of human intrinsic motivation. Some EMIs have explicitly incorporated these constructs, delivering interventions aimed at promoting a sense of agency, building behavioral skills, and fostering social support. A prominent example is A-CHESS (34), which uses self-reported risk factors like depression or sleep problems, and protective factors such as time with family and Alcoholic Anonymous attendance to detect individuals’ status and trigger motivational feedbacks based on each individual’s situation.

Another well-validated theory is Incentive Sensitization Theory (48, 49), which posits that addiction is driven not by pleasure (or “liking”) of a substance but by sensitized dopaminergic systems that amplifies “wanting” in response to drug-related cues. This theory underpins a range of EMI designs that attempt to intervene in moments of cue exposure. For example, one EMI study asked participants to mark personally relevant high-risk locations (i.e., places associated with past craving or substance use), and use GPS data to deliver interventions as users approached those areas (42). Another study developed a context-aware smoking cessation app, in which participants pressed an “I’m smoking” button before they initiated smoking cessation (29, 50). Each button press was logged with location data, which was then treated as a personalized high-risk zone for individual. Once participants initiated their quit attempt, entering one of these high-risk locations for five or more minutes triggered support messages suggesting coping strategies, such as breathing exercises.

These theory-driven EMIs rely on predefined features to monitor, and a predetermined set of interventions associated with such features. While this approach can be useful with its theoretical coherence, it has notable limitations. First, the generalizability across populations is not guaranteed. For instance, craving triggers may vary significantly across age groups (51), sexual orientation (57), and socioeconomic status (31). Furthermore, many addiction theories, such as cue-induced craving related to the Incentive sensitization theory, have been validated primarily in controlled laboratory settings but not yet fully tested in real-world settings. For instance, cue-induced craving has traditionally been studied using static images of substances that lack diverse contexts, thus recent studies are trying to validate cue-reactivity using movies that contain substance uses in rich contexts (52). However, most of the theories for addiction are yet to be validated in real-world settings.

As an alternative, some researchers have adopted data-driven approach to determine both when or which interventions to deliver. These approaches start with empirical data to identify momentary predictors of risk and then use these predictors to build decision models. For example, one research group conducted an EMA study in a population of homeless smokers seeking for smoking cessation treatment (31). Using EMA data collected during the 6 days prior to a scheduled quit attempt, the authors identified six momentary predictors of smoking lapse: urge, stress, alcohol use, social exposure, cigarette availability, and motivation. Then, in a follow-up study, they shifted focus to real-time prediction of post-quit lapse by creating risk scoring using a weighted sum of those six variables (32). When the lapse risk score exceeded a threshold, the system triggered immediate tailored support messages. This algorithm was later implemented in a mobile application Smart-T2 (44). Based on each participant’s EMA responses, the app estimates momentary lapse risk in real time and delivers tailored feedback according to the predicted risk level. For instance, when risk is low, messages focus on maintaining abstinence motivation and providing general cessation guidance; when risk is high, messages target the most salient trigger among the predictors. In a recent RCT (30), participants who used the Smart-T intervention achieved higher abstinence rates than those who used a comparison app that did not provide real-time, automated intervention. Overall, instead of using pre-defined or pre-existing set of features to predict lapse or craving, the research group started from selecting predictors, built their own prediction model, and later developed mobile application that delivers real-time intervention based on participants’ risk status—if high stress, deliver the intervention such as coping strategies. However, even when these data-driven approaches are described as “real-time,” many systems remain EMA-contingent. This creates two practical constraints. First, interventions may be delayed when risk escalates rapidly or when craving occurs without conscious awareness. Second, the approach is ineffective when users do not respond to EMA prompts, which can be common in higher-burden or higher-severity contexts.

This line of research exemplifies how data-driven approach can inform both the timing and content of EMI delivery, offering greater flexibility and personalization than theory-based systems. Nonetheless, current data-driven approaches are not without limitations. A central challenge is the heterogeneity of SUDs—both between individuals and within individuals over time and across contexts (53). With respect to between-person differences, while some individuals experience craving in response to environmental cues, others are primarily triggered by emotional distress or stress. As a result, even well-performing predictive models developed based on the population may lack generalizability across individuals. To address this issue, some studies have incorporated user-defined triggers in EMI. For example, one study asked youth participants to select their top three triggers for use from lists of emotional states, social settings, and activities (54). The resulting EMI used these personalized inputs to deliver motivational messages tailored to the individual’s profile. However, because these triggers were based solely on self-report, their effectiveness and accuracy depended on the individual’s level of insight, which is often compromised in more severe SUDs (14, 55).

Beyond between-person variability, SUDs also involve significant within-person fluctuations across time and context. Craving intensity, for example, may shift as individuals cycle through different stages of addiction such as binge/intoxication, withdrawal/negative affect, and preoccupation/anticipation stages. To account for these dynamics, the concept of a computational fingerprint for addiction—an individualized profile of behavioral and neural signatures—has been proposed as a way to better tailor intervention timing and content (56). Relatedly, EMA studies have consistently demonstrated the importance of within-person variability in substance use, including in smokers (57, 58) and opioid users (59). Specifically, one study found that sexual minority youth smokers exhibited context-dependent smoking motives tied to minority stress, underscoring the need for EMIs that adapt not only to the individual but to their current state and environment (57). Although not yet applied to addiction, algorithms developed for DietAlert, a just-in-time adaptive intervention for weight control, illustrate how predictive learning can be embedded into EMI systems (60). In this design, a predictive learning algorithm estimates lapse risk and identifies the top three contributing factors for the lapse. Based on this information, tailored text-based interventions are delivered to users to help prevent lapses. However, despite these advances, few EMI systems for SUDs currently offer real-time risk detection and intervention delivery.

Taken together, these findings highlight a core limitation of many current EMI systems: reliance on static, one-size-fits-all decision rules that do not fully address the complexity of individual differences and dynamic fluctuations in addiction. To offer more effective intervention, EMI should aim to incorporate both individual- and context-specific patterns of craving and lapse risk, and adaptively deliver personalized interventions in real time.

## Suggestions: data-driven, context-aware craving prediction model

**Figure 1:**
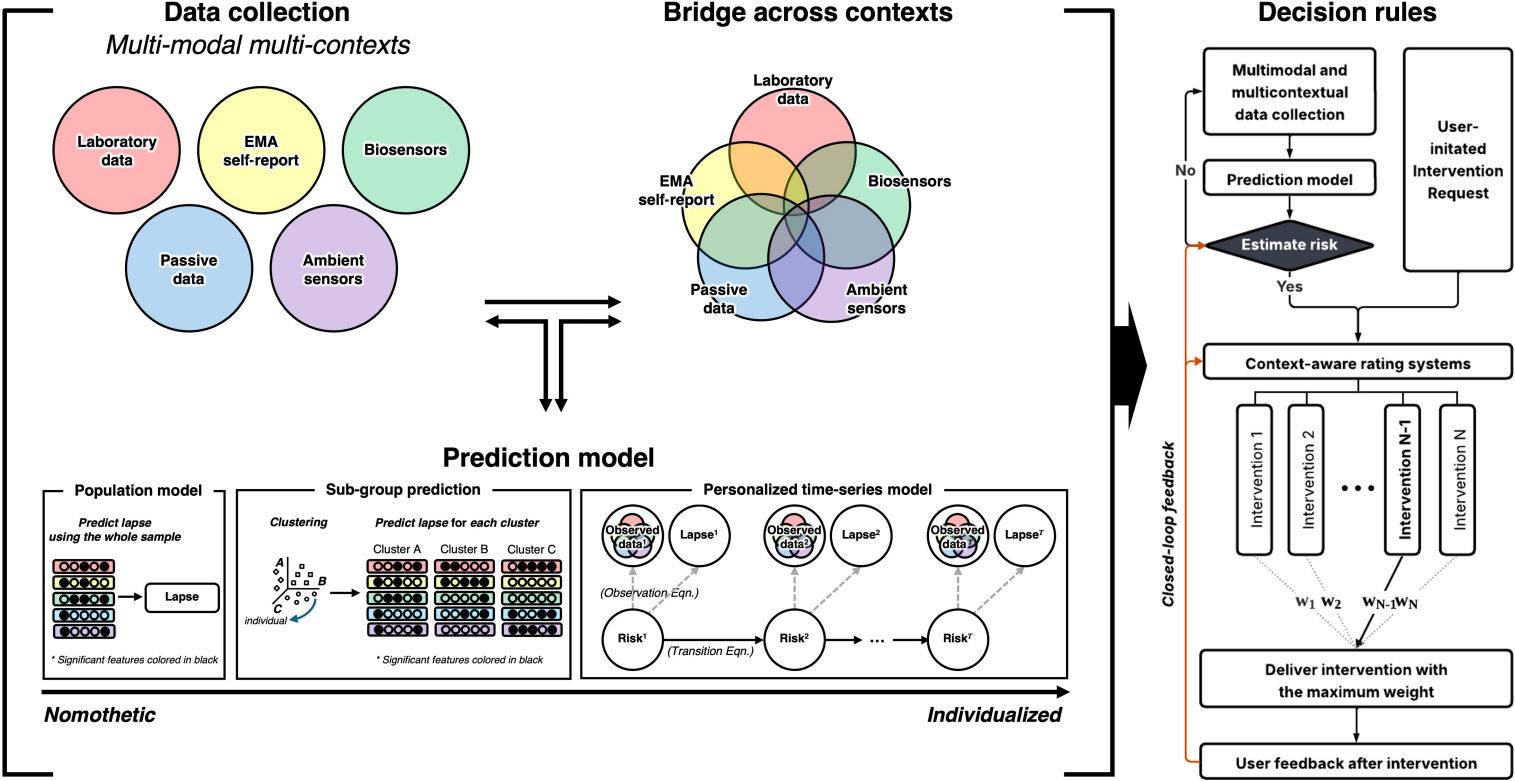
A conceptual framework for building ecological momentary interventions (EMIs). Multimodal data are collected from laboratory tasks, ecological momentary assessment (EMA) self-reports, biosensors, passive data, and ambient sensors. Prediction models—illustrated here with examples ranging from nomothetic to subgroup to individualized approaches—are used to bridge across contexts. After integrating multimodal data, the accumulated knowledge can be applied to refine data collection strategies and enhance prediction models. This data-driven framework can then be implemented in EMIs to support real-time risk estimation, which informs decision rules for intervention delivery. If risk is detected, the system selects the most appropriate intervention based on context; if risk is not detected, users may manually request an intervention when they perceive a need. Following delivery, user feedback is incorporated to recalibrate risk detection and intervention selection, supporting a closed-loop system of continuous personalization and improvement.

We propose a pragmatic, data-driven paradigm for developing personalized EMIs for SUDs, with a specific focus on refining the decision rules that determine both when an intervention should be delivered and which intervention should be selected. To illustrate this approach, we propose a data-driven research protocol for each step of EMI development: data collection, prediction modeling, deciding when to intervene, and selecting which intervention to deliver (**Figure 1**).

### Data collection

The first and most critical step is data collection, as even an optimal model cannot function without robust and representative inputs. The data should be both multimodal and multicontextual. Integrating multimodal data is essential because relying on a single modality inevitably leaves blind spots. As summarized in **Table 3**, EMA, passive data, biosensors, ambient sensors, and laboratory data each provide distinct strengths and limitations. EMA data provides ecologically valid insight into internal states such as craving, mood, and stress, but depends on self-report and is vulnerable to user burden, compliance issues, and response bias (61). Passive data such as GPS, accelerometry, and phone usage can continuously capture behavioral and contextual patterns with minimal user burden, but cannot directly assess psychological states and depends on smartphone use (62, 63). Biosensors yield objective physiological signals, yet can be expensive or uncomfortable and are vulnerable to data loss during charging or non-adherence (64–66). Ambient sensors can help fill data gaps when smartphones or wearables are offline, for example during charging or sleep, by using radio-wave sensing to infer gross movement and respiration (67). Doppler radar, in particular, is often considered relatively privacy-preserving because it does not record audio or video and typically yields derived motion or physiological signals rather than identifiable images. This contactless approach enables continuous monitoring of vital signs without attaching sensors to the body. However, radar-based sensing is generally limited to fixed in-home coverage, and large-cohort deployments remain rare (68, 69). Laboratory data offer high control and precision but lack ecological validity and are temporally and spatially constrained, with many experimental instruments such as functional magnetic resonance imaging (fMRI) or Electroencephalography (EEG) available only in lab settings. No single modality is sufficient, but in combination, these modalities can complement one another and provide a more comprehensive picture of each person’s.

**Table 3:** Strengths and limitations of different data sources.

Modality	Examples	Strengths	Limitations
Ecological Momentary Assessment (EMA)	Self-reported craving, mood, behavior, stress level	- Capture internal states, as self-report provides insight into momentary psychological and contextual experiences. - Ecologically valid because data are collected in real-world settings.	- Burdensome for users, as frequent participant responses are required. - Prone to compliance issues due to skipped prompts and missing data. - Susceptible to bias and inaccuracies inherent to self-report.
Passive data	GPS, phone use, accelerometer, call patterns	- Require no active user input, reducing participant burden. - Enable high-frequency and continuous data collection. - Reflect real-life routines and contextual behavior. - Provide objective measures independent of self-report.	- Data collection is limited during phone inactivity or sleep. - Do not provide direct access to internal states such as emotion or motivation. - Reflect only partial behavioral context, primarily related to smartphone use.
Biosensors	Heart rate variability (HRV), electrodermal activity (EDA), skin temperature, transdermal alcohol sensor	- Provide physiological measures independent of self-report. - Detect rapid physiological changes in real time.	- May reduce adherence due to discomfort or stigma associated with wearing devices. - Risk of data loss if the device is removed or uncharged. - Device battery capacity restricts the duration of continuous monitoring.
Laboratory data	EEG, fMRI, ECG, eye-tracking	- Provide highly precise physiological or neural measurements under controlled conditions. - Experimental control minimizes confounding variables and noise. - Widely used and validated in prior research.	- Poor ecological validity due to artificial laboratory settings. - Constrained by time and location, as data collection is limited to laboratory sessions.
Ambient sensor	Radar sensors, thermal cameras, motion detectors (e.g., in-home)	- Operate non-intrusively without participant burden. - Enable continuous monitoring without active engagement. - Well suited for tracking sleep and activity in natural settings.	- Restricted to fixed environments. - Require installation and maintenance. - Raise privacy concerns in sensitive settings such as the home.

However, multimodal data does not inherently imply multicontextual data. A study may combine EMA, biosensors, and passive data yet still sample only a narrow slice of daily life, for example waking hours or periods of active phone use. Sole reliance on real−world EMA can also miss information collected in the controlled experimental settings. We recommend capturing behavior across a broad range of contexts. In the laboratory, participants can be exposed to alcohol-related cues through images or videos while physiological signals are collected via high-resolution modalities such as fMRI, EEG, Electrocardiography (ECG), Galvanic Skin Response (GSR), or Photoplethysmography (PPG). These data help model how specific cues elicit craving-related responses under tightly controlled conditions. Using cues presented as virtual reality can serve as an intermediate, semi−controlled context that bridges laboratory and real−world environments (70, 71). In naturalistic settings, participants complete EMA surveys while their smartphone and biosensor data are collected to capture day-to-day fluctuations in craving, mood, and behavior. To cover periods when wearable or smartphone−based sensing is unavailable, for example during sleep or charging, ambient radar sensors at home can passively monitor heart rate, respiration, and sleep patterns. Together, these data provide a continuous and context-rich information that connects tightly controlled laboratory responses with everyday fluctuations and offline periods.

After data collection, these multimodal and multicontextual data must be carefully preprocessed, as they differ in format, scale, and temporal resolution. Time-series data should be aligned to a shared time scale, ensuring normalization across sources with different sampling frequencies—for example, synchronizing lab-grade ECG with smartwatch-derived HRV—and standardizing time zones and units. Aligning timestamp is especially critical when combining high-frequency continuous data, such as heart rate, step count, or radar signals, with low-frequency self-report like daily EMA. Time series often need to be summarized into representative features because the sampling frequency of EMA differs from passive data, biosensors, and ambient sensing. For example, variability in time series can be summarized using measures such as standard deviation, root mean square of successive differences (RMSSD), or windowed slopes, with feature choice guided by the research question. When no clear standard exists in the literature, or even when one does, comparing alternative indices through model selection can help identify the most informative features.

Missing data are also common and often systematic in multimodal studies that include EMA and sensors. Researchers should anticipate various forms of missingness, including random nonresponses in EMA, device−related dropout in biosensors, and contextual gaps when participants are asleep or not carrying devices. These patterns can introduce nonrandom missingness that biases model training and reduces generalizability. Thus, appropriate strategies should be applied, ranging from relatively simple methods such as interpolation to more advanced approaches including multiple imputation, inverse probability weighting, and joint modelling (72). Beyond posing analytic challenges, missingness can itself be clinically informative. For example, patterns of nonresponse may reflect disengagement or stress-related dropout, which could be predictive of relapse or heightened craving risk.

### Building prediction models

After collecting and preprocessing the multimodal, multicontextual data, the next step is to translate these inputs into predictive models. The goal is to predict individualized risk that could later enable timely and personalized interventions. There is no single best approach. Below we outline several examples, ranging from simple rule−based classifiers to complex, continuously updated time-series models. Regardless of the specific model, an essential part is to link and validate predictors across different contexts and modalities so that decision policies remain portable and scalable.

A most common approach uses nomothetic, population-level models to predict addiction-related outcomes. These models pool data from many individuals and then predict, for example, each person’s craving risk or relapse. Rich time series are typically summarized into a small set of person−level features, for example mean craving or intraindividual variability, and a single group−level model is trained. Recent studies included dynamic information by looking at how variables change within people over time and modeled within-person relationships using network analysis (73) or hierarchical regression (74). However, these approaches still rely on group−level estimation and require reducing individual time series to a limited set of features, which may obscure individual−specific patterns of risk and addiction−related processes (75).

To improve generalizability while retaining some tailoring, individuals can be classified into subgroups based on behavioral or contextual characteristics, followed by targeted prediction within each group. This remains a nomothetic framework, yet accurate subgrouping can yield more tailored models than fully nomothetic approaches and remain simpler and more interpretable than fully idiographic methods. Unsupervised learning can be applied directly to multi-dimensional real-world data to identify naturally occurring clusters without predefined labels (76–78). Once clusters are formed, a random forest classifier can be trained to predict cluster membership and extract feature importance estimates, highlighting variables that distinguish groups (79). To ensure these subgroup models are useful in practice, validation is a critical step. Temporal cross-validation can be used to assess whether individuals are consistently classified into the same subgroup over time—for example, training on week 1 and testing on week 2. In addition, out-of-sample validation in new participant samples helps establish whether the clustering itself generalizes beyond the original dataset. After validation, subgroup-specific prediction can then be mapped to intervention strategies. For instance, one group may drink alone at home during meals and thus benefit from pre−meal interventions. Another may drink when stressed at home and benefit from stress−triggered interventions. Others may binge in social settings and benefit from event− or location−sensitive interventions. This approach yields semi−personalized intervention that reflect diverse risk pathways. However, even within subgroups, individuals can exhibit unique patterns.

One way to address this limitation is through idiographic approaches that model risk at the level of the individual. A recent example is based on State Space Modeling (SSM), which builds a personalized time-series model for each individual (75). The central assumption is that there is a latent psychological state that cannot be observed directly. The latent state is inferred from observable data through computational modeling, in which mathematical equations represent how observed variables relate to latent states, and parameters are estimated to capture cognitive processes of interest. SSM includes an observation equation that links observed variables, for example EMA responses, to a latent psychological state such as lapse risk, and a transition equation that models how that latent state evolves from one time point to the next given the previous state and time−varying inputs. While offering insight into how a person’s internal risk state changes day by day, or time to time, SSM yielded better predictive accuracy compared to standard models like logistic regression or gradient boosting. Notably, we suggest that contextual variables can also be incorporated in the model. The observation equation could represent how context influences the expression of risk, or the transition equation could represent how context shapes risk trajectories. For example, being at home may amplify craving during periods of loneliness, captured by EMA or passive indicators such as prolonged social media use, while being at work may elevate stress detected via HRV and confirmed by self-report. Moreover, laboratory−derived information can be incorporated as well, informing priors or person−specific parameters. For instance, a stronger physiological response to substance cues can inform higher prior weight on cue−induced craving. Researchers can also quantify an “ability to perceive craving” by comparing self−report with physiological indices, and then adjust the relative weight given to EMA versus physiological signals accordingly. This approach is particularly relevant for addiction treatment, as the ability to perceive craving varies widely across individuals, and people with SUDs often show impaired self-monitoring of craving (80, 81). These examples illustrate how multimodal, multicontextual data can support personalized, context−aware, and dynamic prediction.

Finally, once a model is built, a crucial step is to examine whether the predictors hold across contexts and modalities, because psychological processes in one context are likely to differ from those in other contexts. We therefore propose cross−context validation, which tests whether a feature that predicts risk in one context remains predictive in another, and modality bridging, which identifies practical substitute signals when the original modality is unavailable. For example, if a laboratory cue−exposure task shows that reduced high−frequency HRV predicts cravings, researchers can derive an analogous feature measured via a smartwatch photoplethysmography and test whether it predicts EMA-reported craving in daily life. Comparable validation can extend to offline periods such as sleep using radar−based sensors that infer HRV (82–84). The essential step is to compute comparable features across contexts and modalities, and to verify that they track the same underlying construct. Stress offers another example. In the laboratory, stress can be indexed through subjective ratings or salivary cortisol level, whereas in daily life it can be inferred from HRV patterns, smartphone usage such as screen time or social-app engagement, or EMA. By ensuring consistency in the core features (e.g., HRV, stress) across different modalities and settings, researchers can evaluate whether predictors working in one context (e.g., laboratory) remain predictive in another (e.g., daily life). The cross-context validation and bridging supports robustness of the predictors across contexts. Moreover, it could make the predictors more usable in practice, because validated substitutes keep models working when data sources change or drop out, enabling reliable prediction in the moments and places where people live.

### Deciding when to intervene and which intervention to deliver

Once a generalizable, context-aware prediction model is established, the next step is to translate its outputs into decision rules for when and which intervention to deliver (**Figure 1**). These rules should ensure that intervention is provided at moments when the individual is vulnerable to lapse and receptive to assistance.

The first step is to decide which features to monitor during deployment. This decision can be guided by feature-selection methods from the prediction model, which highlights the strongest predictors of risk. Focusing on these key features during deployment may help keep the system efficient. Efficiency is particularly important in ecological momentary studies because smartphones and wearable devices have limited battery capacity; continuous sensing from GPS, HRV monitors, or accelerometers can quickly drain power, reducing compliance and making long-term deployment impractical if energy use is not managed. Moreover, to function effectively, the model must run continuously on a mobile device or backend server, with active permissions for relevant data sources such as GPS, HRV, and EMA prompts. Near real-time data from these sources are then used as inputs to estimate whether the individual is currently at risk.

Predicted risk scores can be linked to decision policies that vary by context, risk history, or feedback on prior interventions. Critical contexts such as home, workplace, being with friends, being alone, or attending a social event can be identified from model parameters or feature−importance analyses. High-risk contexts can be assigned lower thresholds to increase sensitivity, whereas low-risk contexts can be assigned higher thresholds to reduce unnecessary interventions. To improve accuracy, decision rules can also require converging signals within a short time window. For example, a stress−triggered intervention may be delivered only when high EMA stress ratings are accompanied by an HRV shift and increased social media use. Similarly, a cue−exposure intervention may be triggered when GPS detects proximity to a flagged location along with dwell time or a relevant calendar event. Even when relying on a nomothetic prediction model, tailoring detection to context can improve performance, such as emphasizing loneliness measures when at home or arousal changes when at social gatherings.

Context awareness also benefits the choice of intervention. By identifying the most active risk factors at a given moment, the system can match interventions to the dominant trigger while accounting for feasibility in the current setting. For example, mindfulness exercises may reduce stress but are more practical at home than at a party, while refusal−skills prompts may be better suited to social contexts. Matching intervention can be guided by several criteria that scores each intervention for effectiveness against specific risk factors and for feasibility in different contexts. The system can then select the intervention with the highest combined score for the detected risk profile in the identified contexts.

Because no automated system is perfect, a user−initiated intervention request option should be available to trigger immediate support when users experience intense craving without receiving a suitable intervention from the system. Even in such cases, context awareness can guide the system to select the intervention most appropriate to the user’s current state. This option could further enhance personalization as it provides additional information about when users perceive themselves to be at risk. When a user presses the button, the preceding data can be logged as a confirmed high−risk episode so that future decisions could incorporate such information in detecting risk. We also suggest collecting feedback from users on the effectiveness of the interventions they receive, including both timing and type, so that risk detection and intervention selection can be continuously updated. This process should operate as a closed loop, with ongoing monitoring of user burden, intervention effectiveness, together with periodic retraining or reweighting, so that the system can adapts to the within-person fluctuations characteristic of addiction.

In sum, a well−calibrated prediction model can guide both the timing and content of intervention. Context−aware decision rules, converging signals, and adaptable thresholds help balance sensitivity with user burden, while flexible intervention matching increases the likelihood that support is feasible and relevant in the moment. Incorporating user feedback within a continuous learning loop further enhances personalization, helping to translate predictive modeling into practical, engaging intervention.

## Practical considerations: feasibility, burden, and privacy

Although multimodal, multicontextual EMIs may improve risk detection and intervention matching, their real-world implementation will depend on whether these systems can be deployed and sustained in daily life and routine clinical settings. Accordingly, several practical hurdles should be considered and addressed.

A primary constraint is technological feasibility. Continuous collection of multiple data streams is limited by current technology and infrastructure, including battery life, device reliability, and cost. Battery limitations are a persistent challenge in ecological momentary research because continuous sensing can accelerate power depletion and increase missing data. Device reliability must also be established under naturalistic conditions, including stable connectivity, data completeness, and adequate signal-to-noise ratio. This is particularly important because real-world environments introduce substantial noise and variability that can degrade measurement quality. However, even when a sensing technology is technically capable, it may be impractical for research if it is too expensive to support adequate sample sizes and rigorous validation. Encouragingly, sensing technologies have improved rapidly. Smartwatches illustrate this trajectory: devices that were not widely available two decades ago are now common, include an expanding set of sensors, and continue to improve in battery performance while generally becoming more affordable. These trends suggest that some feasibility barriers may lessen over time.

Participant burden is another major constraint. Intensive monitoring can increase burden and contribute to nonresponse, dropout, and alert fatigue. A pragmatic strategy is stepwise validation. Early studies can recruit participants who are highly motivated to change and willing to engage in higher-intensity monitoring. In many settings, initial work may also focus on lower-severity substances such as nicotine or alcohol, where adherence may be more feasible than in more severe illicit drugs. The purpose of these early phases is to identify a smaller set of high-yield predictors and decision rules that can support a lighter system later, with fewer EMA prompts and fewer required sensors. Notably, participant burden is both a concern for multimodal approaches and a motivation for pursuing them. Many current “real-time” EMIs rely heavily on repeated self-report, which can be particularly demanding for SUD populations. If passive or wearable-derived indicators can reliably substitute for some self-report items, multimodal systems may reduce burden by decreasing reliance on frequent EMA while preserving timely risk detection. For example, smartphone use patterns may provide partial proxies for social context or stress-related states; however, such substitutions require careful validation against self-report and other indicators.

Privacy is equally central for EMIs. Continuous monitoring can raise concerns about privacy, data security, autonomy, and trust in the therapeutic relationship, particularly if monitoring is perceived as surveillance rather than support. These risks require clear safeguards, including transparent consent, plain-language explanations of what each modality captures and what it does not capture, secure data handling, and participant controls to pause sensing, withdraw participation, and request data deletion when feasible. Some modalities require especially careful explanation to prevent misunderstanding. Ambient radar-based sensing does not record audio or video like a camera, but it may still feel intrusive if installed in the home. Passive smartphone sensing can also be misinterpreted as capturing detailed content. For instance, app-use features typically capture which application was used for how long, but they do not collect message content or the specific posts a person viewed. Showing participants concrete examples of data outputs and offering participant-facing dashboards can improve transparency and support informed choice. These, in addition, could encourage motivation of such data collection, as participant could get information about their substance use patterns. Based on the participant-facing dashboards, a policy that acknowledges user’s right to withhold individually produced data and withdrew them from a centralized database could be implemented. But in that instance, the service can notify the user that removing their data can compromise the model’s predictive accuracy for them and even visualize how much the degradation of the accuracy will be.

Taken together, multimodal EMIs are most likely to be clinically useful when they can run reliably over time, remain acceptable to participants, and be governed in ways that preserve privacy and autonomy. Progress therefore requires weighing predictive gains against practical costs, minimizing reliance on high-burden self-report, and implementing monitoring with transparent consent and meaningful user control. We believe that there are clinical contexts in which greater burden, cost, or more intensive monitoring may be justifiable if the expected benefit is substantial and participation is fully voluntary. For example, some individuals at elevated risk of severe outcomes, such as recurrent relapse with medical complications or hospitalization, may prefer higher-intensity support if it meaningfully improves safety and stability. In such cases, patients and families may be willing to accept added effort or expense and a limited increase in data collection in exchange for potential reductions in acute risk, provided that strong safeguards and clear opt-in controls are in place.

## Discussion

While EMIs for SUDs show promise, their effectiveness remains inconsistent. To address this, we shift the question from “whether EMI works” to “when, for whom, and under which decision policies it works.” We argue that decision rules—how the system determines when to intervene and what to deliver—are central to improving effectiveness. We propose a practical pathway toward person- and context-sensitive decision making: collect complementary signals from controlled laboratory tasks, everyday EMA, passive sensing or biosensors, and ambient sensing during offline periods; translate these signals into individualized risk estimates; and link predictions to simple, auditable policies for timing and content. Such policies should emphasize converging cues within short time windows, adaptive thresholds that shift with context and risk history, and lightweight human-in-the-loop safeguards such as a “Help” button and post-intervention feedback.

This review offers a conceptual blueprint for EMIs in SUDs, but translating this blueprint into practice presents challenges. The feasibility of multimodal sensing depends on cost, device availability, battery life, data governance, and also on whether devices are sufficiently comfortable and acceptable to wear over long periods. Building well-performing, context-aware prediction models also requires both computational expertise and deep knowledge from addiction medicine and psychology, underscoring the need for active, multidisciplinary collaboration.

While these steps may not be implemented all at once, EMIs have the potential to narrow the treatment gap if they can learn to identify and respond to the moments that matter. Building straightforward, transparent decision policies on top of multimodal, multicontextual data—and evaluating them in well-designed, real-world studies—could provide a practical route for moving from conceptual promise toward meaningful clinical impact.
